# Flicker‐induced retinal vascular dilation in ipsi‐ and contralateral eyes of patients with carotid stenosis before and after carotid endarterectomy: a prospective study

**DOI:** 10.1111/aos.15107

**Published:** 2022-02-06

**Authors:** Marianne Ala‐Kauhaluoma, Pirkka Vikatmaa, Suvi M. Koskinen, Petra Ijäs, Krista Nuotio, Heli Silvennoinen, Kristiina Relander, Perttu J. Lindsberg, Lauri Soinne, Paula A. Summanen

**Affiliations:** ^1^ Department of Ophthalmology University of Helsinki and Helsinki University Hospital Helsinki Finland; ^2^ Department of Vascular surgery University of Helsinki and Helsinki University Hospital Helsinki Finland; ^3^ Department of Neurology University of Helsinki and Helsinki University Hospital Helsinki Finland; ^4^ Department of Radiology, HUS Diagnostic Center University of Helsinki and Helsinki University Hospital Helsinki Finland; ^5^ Department of Neuropsychology HUS Neurocenter, University of Helsinki and Helsinki University Hospital Helsinki Finland

**Keywords:** dynamic vessel analyzer, carotid stenosis, carotid endarterectomy, flicker‐induced retinal vasodilation

## Abstract

**Purpose:**

Retinal vascular function was assessed in patients with carotid stenosis (CS) before and six months after carotid endarterectomy (CEA) and in controls at a six‐month interval.

**Methods:**

We studied 68 patients (81% male, mean age 69) and 41 healthy non‐medicated controls (77%, 68) from March 2015 to December 2018. Our ophthalmological examination included flicker‐induced arteriolar and venular measurements with a Dynamic Vessel Analyser in both eyes.

**Results:**

At baseline, flicker‐induced arteriolar and venular dilation was reduced in the ipsilateral eyes of the patients compared with dilation in the controls (arteriolar 1.0% versus 2.6%, p = 0.001 and venular 2.2% versus 2.8%, p = 0.049). These differences subsided after CEA. In patients' ipsilateral eyes, flicker‐induced arteriolar dilation was borderline postoperatively (preoperative 1.0% versus postoperative 1.6%, p = 0.06), whereas venular dilation increased (2.2% versus 2.8%, p = 0.025). We found various tentative associations with the change in flicker‐induced dilations after CEA, but not with the preoperative dilations.

**Conclusions:**

Postoperative recovery of the reduced flicker‐induced arteriolar and venular dilatation in the ipsilateral eye shows that, after CEA, the activity‐dependent vascular reactivity of haemodynamically compromised retinal tissue can improve.

## Introduction

The major aetiology of carotid stenosis is atherosclerosis, and the earliest manifestation of an atherosclerotic lesion, endothelial dysfunction, leads to abnormal platelet aggregation and increased vascular permeability (Gimbrone & Garcia‐Cardena 2016), with important implications for regulation of the microcirculation. The retinal microvasculature may reveal pathology in the brain; increased risk of stroke has been linked to smaller arteriovenous ratio (AVR) (Wong *et al*. 2001), to larger venular calibre (Ikram *et al*. 2006), and in patients with type 2 diabetes, also to smaller arteriolar calibre (Klein *et al*. 2007). Characteristics shared by the retinal and cerebral vasculature, ones such as local activity‐dependent autoregulation, provide a compelling opportunity to assess retinal arteriolar and venular microcirculation *in vivo* (Liew *et al*. 2008).

The possibility to measure retinal vessel diameters and reactions has resulted from developments in dynamic retinal vessel analysis (Garhofer *et al*. 2010). A commercially available Dynamic Vessel Analyser (DVA) uses flicker light as a physiological provocation to investigate alterations in retinal vascular diameters (Dorner *et al*. 2003; Garhofer *et al*. 2010). Evidence exists of reduced flicker‐induced retinal vasodilation in diabetes mellitus (DM), hypertension, hyperlipidemia and obesity; all known risk factors for cardiovascular disease (Lim *et al*. 2013). A weak correlation is evident between flicker‐induced retinal vasodilation and brachial artery flow‐mediated dilation (FMD) (Pemp *et al*. 2009). FMD represents the gold standard in clinical assessment of endothelial function in the macrocirculation (Deanfield *et al*. 2007).

After successful carotid endarterectomy (CEA), ocular circulation increases (Geroulakos *et al*. 1996; Riihelainen *et al*.  1997; Wong *et al*. 1998; Cohn *et al*. 1999; Costa *et al*. 1999; Kawaguchi *et al*. 2012). Postoperatively, improvement in neuroretinal function appears in measurements such as the electroretinogram (ERG) (Machalińska *et al*. 2017), visual field (Qu *et al*. 2015) and dark adaptometry (Havelius *et al*. 1997). We are aware of only one report with DVA applied to assess retinal circulation in asymptomatic carotid stenosis (CS) patients undergoing CEA (Machalinska *et al*. 2019), and it found reduced venular, but not arteriolar, vasodilation preoperatively in patients compared with controls, with no change in vascular reactions three months after CEA.

In the current study, we evaluated flicker‐induced retinal vasodilation with DVA. Our hypothesis was that (1) arteriolar and venular flicker‐induced vasodilatation is less pronounced in CS patients before surgery than in controls, and (2) after successful CEA, flicker‐induced retinal vasodilation increases. We also explored the potential associations between vascular reactions and clinical features.

## Methods

### Study design

Our prospective non‐randomized case control study, the Helsinki Carotid Endarterectomy Study‐Brain and Eye SubsTudy (HeCES‐BEST) (Nuotio *et al*. 2018), is a multidisciplinary collaboration including ophthalmologists, neurologists, neuroradiologists, vascular surgeons and neuropsychologists to assess findings in the brain and eye before CEA and six months after CEA. We examined 71 eligible patients and 42 healthy controls between March 2015 and December 2018 in Helsinki University Hospital, Finland, including patients who had CS of 70% or more in their first computed tomography angiography (CTA) evaluation. Part of the data concerning this same cohort has been published earlier (Ala‐Kauhaluoma *et al*. 2020; Ala‐Kauhaluoma *et al*. 2021). We excluded patients with a recent (<6 months) cerebral infarction and excluded the DVA data of participants who had used caffeine or nicotine within 3 hours prior to the examination. Unmedicated age‐ and sex‐matched healthy controls we recruited from senior and exercise clubs, and among hospital staff, relatives and friends. The ethics committee of the Hospital District of Helsinki and Uusimaa approved our study, which complies with the tenets of the Declaration of Helsinki. All participants gave their written informed consent.

### Patients and controls

We examined both eyes of 68 patients after excluding 3 patients: one with recent stroke, one lacking co‐operation during DVA measurements due to light sensitivity and one (who was lost to follow‐up), for caffeine intake and cigarette smoking two hours prior to the DVA examination at baseline examination. After our exclusion of one control with newly diagnosed cancer, 41 controls underwent an eye examination, with a randomly chosen eye serving as the control for the patient's ipsilateral and the other eye as for the contralateral eye.

Patients underwent surgery a median of 2 (range, 1–80) days after baseline examination, the longest delay being due to severe anaemia needing investigation. Altogether, 11 patients (16%) had undergone contralateral CEA at a median of 0.6 (range, 0.2–17.2) years before ipsilateral CEA, and 8 patients (12%) underwent contralateral CEA during follow‐up at a median of 2.0 (range, 1.1–3.7) months after ipsilateral CEA. The second evaluation was done at a median of 5.9 (range, 5.3–8.6) months after ipsilateral CEA. We lost six (9%) patients to follow‐up: three withdrew, two did not respond to our invitation, and one was not invited, because carotid ligation was performed, due to occlusion. Controls we examined twice at a median of 5.9 (range, 4.8–6.9) months interval.

Sources of patient data were clinical records, our research assistants' interviews, a questionnaire completed by the patients and blood samples collected at baseline.

A novel diagnosis of dyslipidemia resulted if low‐density lipoprotein (LDL) level exceeded 3 mmol/l. We measured blood pressure once, immediately before DVA examination, with an automatic sphygmomanometer (Omron, Omron Healthcare, Kyoto, Japan), while the patient was in a sitting position. We calculated mean arterial pressure (MAP) with the formula: MAP = [Diastolic blood pressure (DBP)] + 0,412 × [Systolic blood pressure (SBP) – DBP] (Papaioannou *et al*. 2016).

Table 1 shows that patients and controls were similar regarding age, gender, SBP and triglyceride concentrations. Differences between the two groups were in regard to smoking, BMI, DBP, MAP, and to level of cholesterol, high‐density lipoprotein (HDL) and LDL. Of all patients, 63 (93%) used statins. Symptoms related to CS included amaurosis fugax (Afx) in 28 (41%), hemispheric TIA in 17 (25%), stroke (six months earlier) in one (1.5%), neovascular glaucoma (NVG) in one (1.5%), branch retinal artery occlusion (BRAO) in one (1.5%) and ocular ischaemic syndrome (OIS) with reduced VA in one (1.5%); 19 (28%) were asymptomatic.

**Table 1:** Baseline characteristics in patients with carotid stenosis versus controls.

	Patients	Controls	p‐value
	N = 68	N = 41	
Age, years	69 (7)	68 (5)	0.40*
Gender, male	55 (81%)	31 (76%)	0.51**
Smoking	23 (34%)	2 (5%)	<0.001***
Diabetes mellitus	22 (32%)	0	<0.001***
Systemic hypertension****	57 (84%)	0	<0.001**
Dyslipidemia	64 (94%)	33 (80%)	0.025**
Coronary artery disease	22 (32%)	0	<0.001**
BMI, kg/m	28 (5)	25 (3)	0.002*
SBP, mmHg	142 (20)	148 (17)	0.09*
DPB, mmHg	78 (12)	85 (10)	<0.001*
MAP, mmHg	104 (13)	111 (12)	0.006*
Cholesterol, mmol/L	3.8 (1.1)	5.4 (0.9)	<0.001*
HDL, mmol/L	1.3 (0.4)	1.6 (0.5)	0.001*
Triglycerides, mmol/L	1.3 (0.6)	1.2 (0.8)	0.70*
LDL, mmol/L	2.2 (1.1)	3.6 (0.7)	<0.001*

Data presented in mean (SD) or number (%).

BMI = body mass index, DBP = diastolic blood pressure, HDL = high‐density lipoprotein, MAP = mean arterial pressure, LDL = low‐density lipoprotein, SD = standard deviation, SBP = systolic blood pressure.

*Two‐sample*t*‐test.

**Chi‐square test.

***Fisher's exact test.

****Systemic hypertension diagnosed and medicated.

We analysed the grade of CS according to the North American Symptomatic Carotid Endarterectomy Trial (NASCET) method with CTA (Barnett *et al*. 1991). A subtle near‐occlusion (NO‐s) we defined as a decrease of 1.0 mm in distal luminal diameter beyond a tight stenosis; in full collapse of near‐occlusion (NO‐fs), the luminal reduction exceeded 2.5 mm (Koskinen *et al*. 2017; Meershoek *et al*. 2018). Ipsilateral CS was <70% in 6 patients (9%), 70%–99% in 28 (41%), NO‐s in 27 (40%), NO‐fs in 6 (9%) and totally occluded in one (1%). Contralateral CS was <70% in 55 patients (81%) and 70 to 99% in 7 (10%), NO‐s in 2 (3%), NO‐fs in one (1%) and totally occluded in 3 (4%). We used carotid ultrasonography to rule out CS in our controls.

### Ophthalmological examination

We conducted comprehensive eye examinations including best‐corrected visual acuity (BCVA) obtained with ETDRS charts scored in logMAR units and intraocular pressure (IOP) measurement with a rebound tonometer (Icare TA01i Tonometer, Icare Finland OY, Vantaa, Finland).

After pupillary dilation with tropicamide, we performed anterior and posterior bio‐microscopy. We utilized a Zeiss FF450^plus^ fundus camera (Carl Zeiss, Jena, Germany) to take 30° and 50° colour and red‐free fundus photographs. A semi‐automated software Vesselmap and VISUALIS (Imedos Systems Ltd, Jena, Germany) allowed us to measure central retinal arteriolar equivalent (CRAE), central retinal venular equivalent (CRVE) and AVR; we aimed to use the six largest arterioles and venules (Knudtson *et al*. 2003). Ocular signs of CS we divided into embolic (Hollenhorst plaques and BRAO) and hypoperfusion‐related (midperipheral haemorrhages) findings, the former was found in 4 and the latter in 13 patients (Ala‐Kauhaluoma *et al*. 2021). Enhanced‐depth spectral‐domain optical coherence tomography (EDI‐OCT) (Heidelberg Spectralis; version 6.3.2.0; Heidelberg Engineering, Heidelberg, Germany) allowed us to measure subfoveal choroidal thickness (SFCT) (Ala‐Kauhaluoma *et al*. 2020).

### Dynamic vessel analyser

DVA (Imedos GmbH, Jena, Germany) served to determine retinal vessel diameters from video sequences obtained with a conventional camera; this system is described in detail elsewhere (Garhofer *et al*. 2010). Our study protocol started and ended with 50 s of steady illumination; and, in between, three circles with 30 s of flickering light at 12.5 Hz frequency and 50 s of steady illumination. We chose a superior or inferior temporal arteriole and venule segment around 0.5 DD in length 1–2 DD from the optic nerve. Sites where a venule and arteriole appeared close to each other, or vessels with diameter <90 μm we avoided. DVA software automatically overlaid three flicker cycles and displayed a summary profile showing vessel‐diameter changes in percentages relative to the baseline. If a good summary profile was not achievable in at least two flicker cycles, we excluded the DVA data from further comparison.

### Statistical analysis

We tested continuous normally distributed variables with two‐sample t‐test (presented as means and standard deviations). Continuous non‐normally distributed variables we tested with the Wilcoxon signed‐rank test for related samples and the Mann–Whitney *U‐*test for non‐related samples (medians and interquartile ranges). Chi‐square or Fisher's exact tests allowed us to test categorical variables (frequencies and percentages). We analysed associations and correlations of categorical characteristics with flicker‐induced retinal vasodilation at baseline, and the change in them postoperatively or at follow‐up. Tests for associations were the Mann–Whitney *U* and Kruskal–Wallis, and correlations were by Spearman correlation coefficients. Statistical analyses were done using SPSS for Windows (version 26, IBM, Armonk, NY). Statistical significance we set at 0.05.

## Results

Patients and controls presented, both pre‐ and postoperatively, with normal median BCVA (logMAR 0 or better) and normal mean IOP (from 13 to 15 mmHg) (data not shown).

### Median flicker‐induced retinal vasodilation

**Figure 1:**
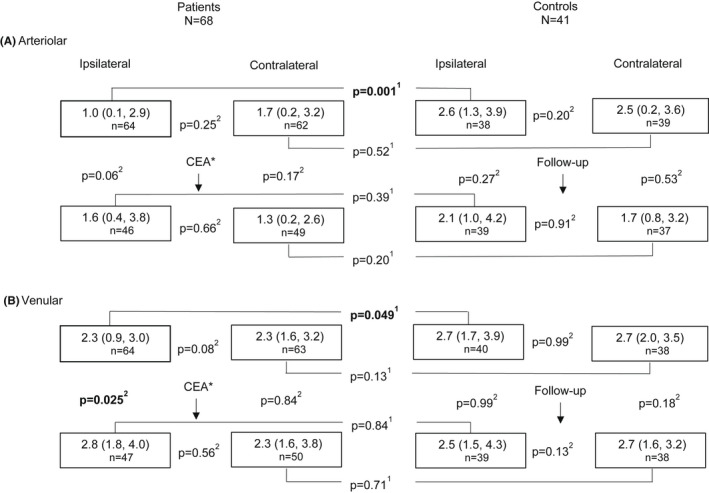
Median (IQR) flicker light‐induced retinal arteriolar (A) and venular (B) dilation (%) in ipsi‐ and contralateral eyes of patients before and 6 months after carotid endarterectomy (CEA) compared to controls at a six‐month interval. Dynamic Vessel Analyzer (DVA) shows vessel diameter changes in percentages relative to the baseline. *Postoperative data without eight patients who underwent CEA of the contralateral side during follow‐up. n= number of acceptable DVA measurements; ^1^Mann‐Whitney U test; ^2^ Wilcoxon signed‐rank test.

Preoperatively, arteriolar and venular dilations were reduced in the ipsilateral eyes of the patients compared with dilations in the controls (median 1.0% versus 2.6% and median 2.2% versus 2.8%, p = 0.001 and p = 0.049, Mann–Whitney *U*‐test, Fig. 1). Arteriolar and venular dilations were similar between the ipsi‐ and contralateral eyes of the patients and between the eyes of the controls.

Postoperatively, arteriolar dilation showed a trend towards an increase in the patients’ ipsilateral eyes (median 1.0% versus 1.6%, p = 0.06, Wilcoxon signed‐rank test, Fig. 1), but remained unchanged in the patients' contralateral eyes, and at follow‐up in the eyes of the controls. After CEA, venular dilation increased in the patients' ipsilateral eyes (2.3% versus 2.8%, p = 0.025), but remained unchanged in the patients' contralateral eyes, and at follow‐up in the eyes of the controls. Postoperatively, arteriolar or venular dilations in patients did not differ from dilations in the follow‐up examinations of the controls.

When we excluded 13 contralateral eyes of patients with contralateral CS ≥70%, we found one change compared with data in Fig. 1; preoperative venular dilation was decreased in the ipsi‐ versus contralateral eyes of the patients (median 2.3%, IQR 0.9–3.0, n = 52 versus median 2.4%, IQR 1.6–3.2, n = 51, p = 0.048, Wilcoxon signed‐rank test).

### Associations with flicker‐induced retinal vasodilation preoperatively and with change in dilation postoperatively

Preoperatively, smoking, DM, systemic hypertension, dyslipidemia, levels of lipoproteins, coronary artery disease (CAD), ocular signs of CS, symptoms related to CS and grade of CS, age, body mass index (BMI), SBP, DPB, MAP, AVR, CRAE, CRVE or SFCT showed no associations with retinal vasodilation in the patients. In the controls, we found an inverse correlation between venular dilation and age in the ipsilateral (*r* = −0.32; p = 0.046, Spearman rank correlation), but not in the contralateral eyes (*r* = −0.09; p = 0.61).

**Figure 2:**
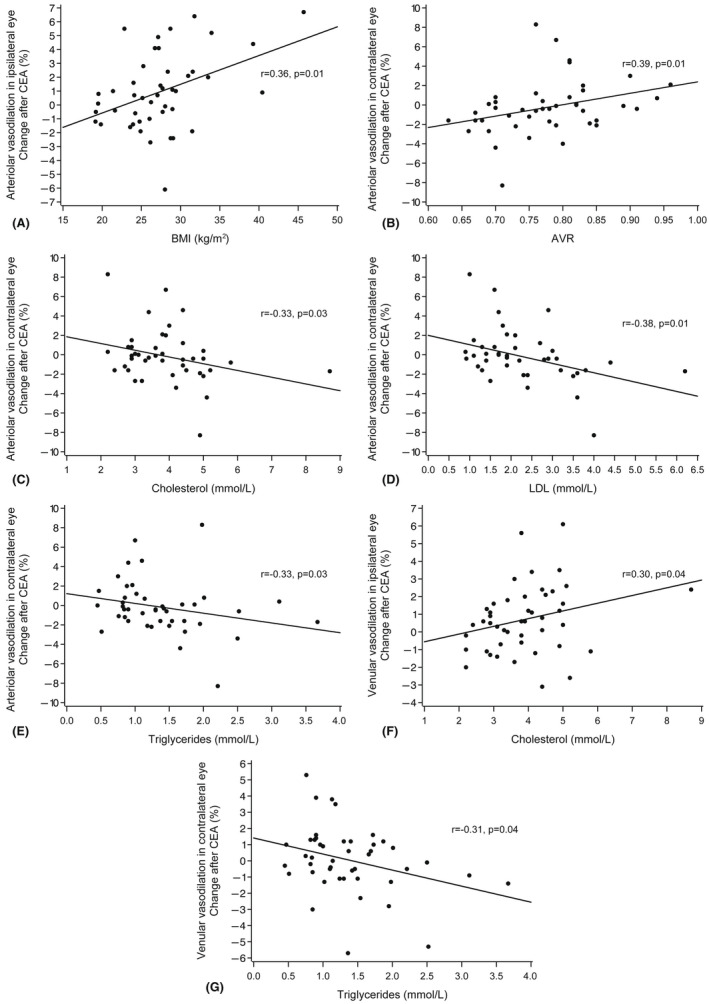
Scatter plot displaying the correlation between clinical features and the change (%) in (flicker‐induced) arteriolar or venular dilation 6 months after CEA in ipsi‐ or contralateral eyes of patients. Postoperative data without eight patients who underwent CEA of the contralateral side during follow‐up. AVR = arterio‐venous ratio, BMI = body mass index, LDL = low‐density lipoprotein.

Patients with CAD showed decreased venular dilation postoperatively in the ipsilateral eyes (p = 0.004, Mann–Whitney *U*‐test) and increased arteriolar dilation postoperatively in the contralateral eyes (p = 0.008, Table 2). Patients with systemic hypertension had decreased arteriolar dilation postoperatively in the contralateral eyes (p = 0.018), and the same occurred in patients with symptoms related to CS (p = 0.001). In all patients, we found a positive correlation between change in arteriolar dilation in the ipsilateral eyes and BMI (*r* = 0.36; p = 0.014, Spearman rank correlation) and in the contralateral eyes and AVR (*r* = 0.39; p = 0.010, Fig. 2).

**Table 2:** Clinical associations with the change in flicker‐induced arteriolar and venular vasodilation 6 months after carotid endarterectomy (CEA) in ipsi‐ and contralateral eyes in 68 patients with carotid stenosis (CS).

	Arteriolar vasodilation						Venular vasodilation					
	Ipsilateral			Contralateral			Ipsilateral			Contralateral		
	n	Change, %	p	n	Change, %	p	n	Change, %	p	n	Change, %	p
Smoking												
Yes	13	1.1 (−0.4, 4.5)	0.20*	13	−0.1 (−1.8, 1.6)	0.90*	13	1.2 (−0.1, 2.7)	0.08*	14	−0.9 (−2.8, 1.2)	0.12*
No	32	0.6 (−1.4, 2.1)		31	−0.4 (−1.6, 0.7)		33	0.4 (−1.1, 1.5)		32	0.1 (−0.7, 1.2)	
Diabetes												
Yes	15	1.6 (−0.5, 5.2)	0.09*	17	−1.1 (−1.9, 0.2)	0.28*	15	0.4 (1.2, 1.6)	0.64*	17	1.0 (−1.0, 1.6)	0.08*
No	30	0.2 (−1.3, 1.6)		27	−0.1 (−1.7, 1.2)		31	0.6 (−0.7, 2.0)		29	−0.4 (−1.1, 0.8)	
Systemic hypertension												
Yes	37	0.7 (−1.1, 2.6)	0.94*	36	−0.6 (−2.1, 0.1)	0.018*	38	0.5 (−0.7, 1.9)	0.74*	38	0.1 (−1.1, 1.2)	0.45*
No	8	0.7 (−1.1, 2.1)		8	1.1 (−0.4, 4.0)		8	0.9 (−1.1, 2.2)		8	−0.3 (−1.3, 1.0)	
Dyslipidemia												
Yes	43	0.7 (−1.0, 2.4)	0.88*	43	−0.4 (−1.7, 0.7)	0.40*	44	0.6 (−0.7, 2.0)	0.66*	44	−0.5 (−1.1, 1.2)	0.53*
No	2	2.1 (−1.4, −)		1	1.5		2	0.1 (−1.0, −)		2	−2.4 (−5.7, −)	
Coronary artery disease												
Yes	14	0.9 (−1.1, 1.6)	0.93*	11	0.8 (−0.3, 4.6)	0.008*	14	−0.2 (−1.3, 0.7)	0.004*	13	−0.5 (−1.3, 1.1)	0.53*
No	31	0.5 (−1.2, 2.8)		33	−0.6 (−2.1, 0.1)		32	1.2 (0.1, 2.4)		33	0.2 (−1.0, 1.2)	
Symptoms related to CS												
Yes	30	0.8 (−1.2, 3.2)	0.52*	29	−1.2 (−2.1, 0)	0.001*	31	0.5 (−1.0, 1.6)	0.79*	31	−0.1 (−1.1, 1.0)	0.74*
No	15	0.7 (−0.5, 1.4)		15	0.3 (−0.4, 4.4)		15	0.6 (−0.6, 2.0)		15	0.3 (−1.1, 1.3)	
Ocular signs of CS												
Plaque related	3	2.0 (−2.4, −)	0.85**	2	−2.3 (−3.4, −)	0.09**	3	0.6 (−1.2, −)	0.59**	2	−1.6 (−3.0, −)	0.31**
Hypoperfusion related	7	0.9 (−1.6, 1.1)		8	0.2 (−0.3, 3.5)		7	0 (−1.7, 1.8)		8	1.1 (−0.5, 1.3)	
None	35	0.7 (−1.0, 2.4)		34	−0.6 (−1.8, 0.8)		36	0.7 (−0.5, 2.1)		36	−0.3 (−1.1, 1.0)	
Grade of CS***												
< 70%	3	0.7 (−0.6, −)	0.24**	38	−0.5 (−1.8, 0.9)	0.28**	3	0.9 (0.4, −)	0.64**	40	0.1 (−1.1, 1.2)	0.31**
70–100%	20	−0.2 (−1.8, 1.9)		4	−0.3 (−1.3, −0.1)		21	0.6 (−0.7, 1.7)		4	0.5 (−1.8, 3.2)	
Near‐occlusion	22	1.0 (−0.3, 3.1)		2	−2.2 (−4, −)		22	0.6 (−1.0, 2.2)		2	−1.8 (−2.9, −)	

Data presented in median (IQR).

n = number of Dynamic vessel analyser measurements.

*Mann–Whitney *U‐test*.

**Kruskal–Wallis test.

***Grade of CS is based on the North American Symptomatic Carotid Endarterectomy Trial (NASCET) method.

In the contralateral eyes of patients, change in arteriolar dilation seemed negatively correlated with total cholesterol (*r* = −0.33; p = 0.031), LDL (*r* = −0.38; p = 0.013) and triglycerides (*r* = −0.33; p = 0.033). Associations with arteriolar dilations occurred neither in the ipsilateral eyes of patients, nor in the controls. Change in venular dilation appeared to have a weak positive correlation with total cholesterol in the ipsilateral eyes of patients (*r* = 0.30; p = 0.041), and a negative correlation with triglycerides in the contralateral eyes of patients (*r* = −0.31; p = 0.038), but this was not evident in the controls.

## Discussion

To our knowledge, this is the first study to show (1) reduced flicker‐induced dilation in both retinal arterioles and venules in the ipsilateral eyes of CS patients compared with dilation in controls before CEA, (2) this difference subsiding 6 months after CEA and (3) an increase in venular reactions after CEA. These postoperative findings indicate the capacity of retinal vascular reaction to improve after CEA. Change in retinal vasodilation after CEA was associated, mostly negatively, with CAD, systemic hypertension, AVR and lipoprotein concentrations; more commonly this association occurred in the contralateral rather than the ipsilateral eye and more commonly in the flicker‐induced arteriolar rather than venular dilations.

Retinal circulation, which is under local autoregulation and mediated by several vasoactive agents, aims to maintain adequate blood flow when physiological conditions (blood pressure and blood glucose concentration) and local metabolic demands vary (Riva *et al*. 1981; Kur *et al*. 2012; Newman 2013). We assume that microvascular function, including neurovascular coupling, may be restricted in patients with CS due to their reduced ocular blood flow. Machalinska *et al*. found preoperative flicker‐induced venular dilation to be reduced in both the ipsi‐ and contralateral eyes of patients compared with their controls (Machalinska *et al*. 2019). We, however, confirmed this only in the ipsilateral eyes when compared to controls, and both in the arteriolar and venular reactions (Fig. 1). In our patients, high‐grade stenosis (≥70%) occurred in 91% in the ipsi‐ compared with 19% in the contralateral side. After excluding 13 contralateral eyes with contralateral CS ≥70%, we observed decreased preoperative flicker‐induced venular dilation in the ipsi‐ versus contralateral eyes. It seems that the microvascular dysfunction in CS patients is dependent on the grade of CS.

Removal of atheromatous plaque in CEA prevents mechanical hindrance of blood flow and allows ocular blood flow to increase, depending on the capacity of retinal autoregulation. Such autoregulation is based on the function of the neurovascular unit: endothelial cells, neurons, glias, pericytes and smooth muscle cells (Kugler *et al*. 2021). Machalinska *et al*. found no increase in flicker‐induced reactions 3 months after CEA, and their conclusion was that ‘microvascular dysfunction is long‐lasting’. On the contrary, we found improvement in the flicker‐induced venular vasodilation in the ipsilateral eyes 6 months after CEA. In line with that Machalinska group study, we, too, observed lack of dilation in the arterioles. The capacity of the arterioles to react is reduced by arteriolosclerosis, accompanied by thickening of the arterial wall and thinning of smooth muscle, these giving way to fibrosis (Blevins *et al*. 2021). While higher AVR indicates less arteriolosclerosis, we observed a greater change in arteriolar dilation after CEA to correlate, only in the contralateral eyes, with higher AVR. All in all, both arteriolar and venular reactions became comparable with reactions in the controls postoperatively.

Our patients had several known risk indicators for endothelial dysfunction. Whether flicker‐induced retinal vasodilation measures endothelial function, as earlier proposed (Lim *et al*. 2013), is unclear. It is worthwhile to notice that we found associations only with the change in flicker‐induced reactions occurring after CEA, but not with their preoperative values. Arterial hypertension, both untreated and treated, has led to reduced flicker‐induced arteriolar reactions (Nagel *et al*. 2004; Peregud‐Pogorzelska *et al*. 2020), and this has been apparent also in patients with CAD (Al‐Fiadh *et al*. 2015). Although we found no correlation with blood pressure values, we did observe an association between decreased arteriolar dilation postoperatively in patients with systemic hypertension (diagnosed and medicated), and in patients with CAD, but both findings only occurred in the contralateral eyes. In our patients with CAD, this finding was true also in the venular reactions in the ipsilateral eyes.

Obese patients have shown reduced arteriolar dilation compared with dilation in the non‐obese (Kotliar *et al*. 2011). We found only a positive correlation with BMI in ipsilateral eyes of patients, which may be due to chance variation as it was not supported by our other observations.

We also studied the correlation between retinal microvascular function and hyperlipidemia, which proved to be complex. The Reimann group (Reimann *et al*. 2009) found in their hypercholesterolemic patients that flicker‐induced vasodilation in both retinal arterioles and venules was reduced. When treated once with LDL apheresis, however, the dilation in retinal venules improved (p = 0.013). Evidence also exists that microvascular function and oxidative stress are linked (Shokr *et al*. 2020), and this linkage is, in part, hyperlipidemia‐mediated.

In our study, the reactions in the contralateral eye were as expected; a smaller change in arteriolar dilation after CEA correlated with higher concentrations of total cholesterol, LDL and triglycerides, whereas in the ipsilateral eye greater change in venular dilation after CEA correlated with higher concentrations of total cholesterol. Treatment by statins may be a confounder: excluding our four patients for whom a statin was prescribed within 2 weeks before CEA from postoperative data made every correlation between lipoprotein concentrations and retinal reactions disappear. This finding may be attributable to small sample size, or to the beneficial effect of statins on retinal microvascular function; the latter has been shown (Terai *et al*. 2011). Our controls showed no correlation between lipoprotein concentrations and flicker‐induced reactions, even though they had higher total cholesterol and LDL concentrations than did the patients (Table 1).

In this same study group, we have confirmed bilaterally thinner SFCT before and after CEA than in controls (Ala‐Kauhaluoma *et al*. 2020). Furthermore, we have found ocular signs of CS, indicating ocular hypoperfusion, as being common in CEA patients (Ala‐Kauhaluoma *et al*. 2021). Neither SFCT nor ocular signs of CS were associated with flicker‐induced vasodilation. Our patient with total carotid stenosis having ocular ischaemic syndrome was an outlier with preoperative (no postoperative visit) flicker‐induced arteriolar and venular dilation (−0.9% and −0.9%). Unfortunately, our one patient with neovascular glaucoma and another with ocular hyperperfusion one week after CEA were unmeasurable with DVA due to poor pupillary dilation.

In our controls, a negative correlation appeared between flicker‐induced venular dilation and age. Likewise, one study with healthy volunteers revealed an age‐related decline in flicker‐induced arteriolar amplitude (Kneser *et al*. 2009). Our controls, however, had a much higher mean age and a narrower age range than did patients of the Kneser group.

The explorative nature of our study allowed us to test associations widely, but the value of these observations is limited by our small sample size. The latter remains the main limitation of our study. Moreover, all of our patients were Caucasian, and 81% was male. Male preponderance is at least partly due to gender differences in incidence and therapy in CS patients (Stoberock *et al*. 2016). A larger prospective study could reveal, (1) whether assessment of retinal vascular reactions can serve as a biomarker of CS patients undergoing CEA, and, in particular, (2) whether poor flicker‐induced reactions can indicate patients at risk of postoperative cerebral or ocular hyperperfusion syndrome.
